# IPF-RAG: temporal-aware retrieval-augmented generation for idiopathic pulmonary fibrosis care

**DOI:** 10.3389/fdgth.2026.1883739

**Published:** 2026-10-14

**Authors:** Xuejia Zheng, Xiang Rui, Teng Long, Liwen Wang, Zhongdong Lv, Jincheng Wang, Zhe Jin, Wendi Huang, Xingbo Dong, Qingwen Wang, Yong Dai

**Affiliations:** 1 The First Hospital, Anhui University of Science & Technology, Huainan, China; 2 Anhui Provincial International Joint Research Center for Advanced Technology in Medical Imaging, Anhui University, Hefei, Anhui, China; 3 Shantou University Medical College, Shantou, China; 4 Department of Respiratory and Critical Care Medicine, Peking University Shenzhen Hospital, Shenzhen, China; 5 Departments of Nephrology, Shenzhen People’s Hospital, Shenzhen, China; 6 School of Medicine, Anhui University of Science & Technology, Huainan, China; 7 Anhui Provincial Academician Workstation of Anhui University of Science & Technology for Autoimmune Disease Diagnostic Technology, Huainan, Anhui, China

**Keywords:** clinical decision support, digital health, electronic health records, idiopathic pulmonary fibrosis, large language models, retrieval-augmented generation, temporal retrieval

## Abstract

Personalized clinical management of idiopathic pulmonary fibrosis (IPF) is hindered by its complex longitudinal course and fragmented, unstructured data. Although large language models (LLMs) and retrieval-augmented generation (RAG) show promise in clinical decision support, current methods face challenges in addressing dynamic disease progression due to static knowledge corpora, a lack of temporal awareness, and susceptibility to semantic noise and hallucinations. To address this, we propose IPF-RAG, a temporal-aware RAG framework for longitudinal IPF care. Driven by a curated dataset of 58 patient trajectories, the framework comprises two core modules. The Temporal Retrieval Module augments semantic matching with temporal-aware evidence anchoring, establishing temporal correlations between disease progression and medical orders. Subsequently, the Structured Enhancement Module integrates this temporal knowledge into structured prompts, applying medical logic constraints to guide LLMs toward evidence-based reasoning. Experiments on internal and external datasets, evaluated by automated assessments and clinical experts, demonstrate that IPF-RAG improves the quality of LLM-generated treatment plans across multiple dimensions. These findings indicate that the proposed approach effectively assists LLMs in clinical decision-making for complex chronic diseases.

## 1 Introduction

Idiopathic pulmonary fibrosis (IPF) is a chronic, progressive interstitial lung disease (ILD) characterized by irreversible scarring and functional decline, with a median survival of only 3–5 years after diagnosis (1–3). Despite advances in understanding its pathophysiology, IPF remains incurable. Clinicians encounter persistent challenges in predicting disease progression and tailoring treatment, largely due to the complexity of longitudinal patient trajectories and the scarcity of integrated knowledge bases (4–6). Crucially, real-world IPF monitoring is often hindered by highly fragmented clinical data that range from asynchronous narrative notes to raw radiological images awaiting expert interpretation, a factor that complicates continuous disease tracking. These multifaceted difficulties result in diagnostic delays and suboptimal management, particularly in healthcare systems with limited specialist resources (3, 7).

Traditional computational approaches, such as rule-based decision support (8) and machine learning models trained on static datasets (9), have attempted to address these gaps yet often prove insufficient (10). They fail to capture the dynamic nature of disease progression or incorporate continuously evolving medical knowledge (11). In this context, Large Language Models (LLMs) have emerged as powerful tools in medicine (12–14), offering promising applications in clinical decision support (15–19). Yet, despite their potential, LLMs face two fundamental limitations in specialized domains such as IPF. First, their reliance on static training corpora introduces an inherent temporal knowledge cutoff, leaving them unaware of the latest antifibrotic drugs or updated clinical guidelines (20, 21). Second, their generalist pre-training often lacks sufficient domain-specific expertise, reducing their capacity to discern subtle longitudinal disease phenotypes (22–24).

Retrieval-Augmented Generation (RAG) offers a natural mechanism to address these gaps by grounding LLM outputs in verifiable, continuously updated external databases (25–30). However, applying RAG to the IPF domain reveals distinct technical hurdles. Clinical data exhibit high heterogeneity, and Electronic Health Records (EHRs) typically store isolated, highly unstructured notes that obscure the chronological progression of the disease (31). Conventional RAG frameworks based on static semantic matching often retrieve anachronistic or redundant semantic noise, failing to align evidence with the dynamic stages of IPF. This leads to two critical challenges: (I) *How can longitudinal clinical data be structured and retrieved to preserve temporal disease trajectories rather than isolated snapshots?* and (II) *How can the retrieved historical evidence be structurally constrained to enforce rigorous medical logic and mitigate hallucination risks during LLM generation?*

To address these challenges, we propose *IPF-RAG*, a temporal-aware RAG framework tailored specifically for longitudinal IPF care, as comparatively illustrated in Figure 1. Based on retrospective data from 2,265 ILD patients, we constructed an IPF dataset that explicitly encodes the time-stamped clinical trajectories of 58 enrolled patients, alongside a pharmacological database of 72 pulmonary fibrosis-related drugs. For Challenge (I), IPF-RAG introduces a Temporal Retrieval Module (TRM) that systematically connects progress notes with corresponding medical orders via explicit temporal associations, replacing naive semantic matching with temporal-aware evidence localization. For Challenge (II), we designed a Structured Enhancement Module (SEM) that incorporates the temporally retrieved knowledge into structured few-shot prompts, acting as a logical constraint to guide LLMs toward evidence-based reasoning. The synergistic operation of these dual modules enables the generation of clinically coherent and personalized treatment plans.

**Figure 1:**
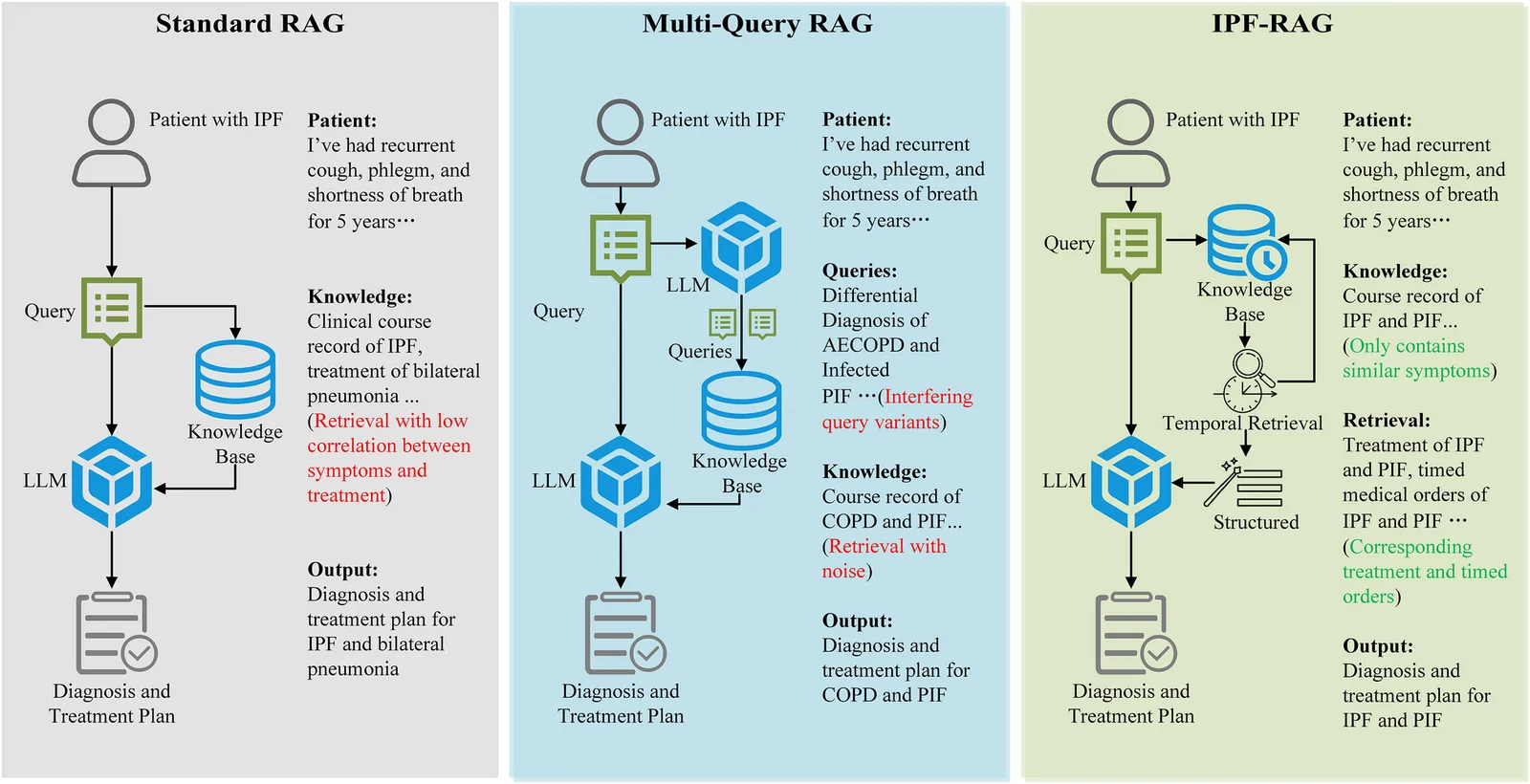
Comparison of Different RAG Frameworks. **(a)** Standard RAG uses a single query for retrieval; **(b)** Multi-query RAG expands the initial prompt into multiple queries; **(c)** IPF-RAG (Ours) integrates temporal retrieval and knowledge structuring to capture time-sensitive information and organize retrieved data systematically.

The main contributions of this paper are summarized as follows:

- We constructed an IPF dataset that captures longitudinal clinical trajectories, shifting the paradigm from static patient snapshots to dynamic, temporal-aware medical retrieval.
- We developed the model-agnostic IPF-RAG framework. Its core Temporal Retrieval and Structured Enhancement modules work together to reduce retrieval noise and maintain strict medical logic.
- We validated the effectiveness of our framework through both automated and expert human evaluations. The results demonstrate that IPF-RAG improves the quality of diagnostic and therapeutic regimens generated by LLMs across multiple dimensions.

## 2 Materials and methods

### 2.1 Dataset and knowledge base construction

#### 2.1.1 Data collection

To address the scarcity of systematically curated IPF datasets, we constructed a structured dataset from real-world clinical records. This retrospective study was conducted in accordance with the Declaration of Helsinki and was approved by the Research Ethics Committee of Peking University Shenzhen Hospital (Approval No. BDSM-IRB (R) [2025] No. 229). The requirement for informed consent was waived by the ethics committee.

We initially identified 2,265 patients with interstitial lung disease (ILD) from the hospital database between 2014 and 2024. The de-identified research dataset was accessed and analyzed after ethics approval was granted. To identify IPF cases, we applied international diagnostic guidelines (32) and included patients whose high-resolution computed tomography (HRCT) findings were consistent with usual interstitial pneumonia (UIP) or probable UIP. Patients with alternative causes of ILD were excluded where relevant according to the available clinical records. Raw clinical narratives were converted into structured text files while preserving the chronological sequence of clinical events. Direct identifiers were removed before analysis, and each subject was represented by a study-specific identifier. The final dataset comprised 58 curated IPF clinical trajectories, totaling more than 470,000 words.

#### 2.1.2 Data structuring and preprocessing

To reduce noise in the raw EHRs, we implemented a hybrid chunking algorithm combining structural parsing with semantic segmentation. We formalized the processed data using the relational schema shown in Equation 1:

R(Patient_ID,Timestamp,Record_Type,Content). (1)

Here, *Patient_ID* serves as the primary key, *Timestamp* tracks the exact event timing, and *Record_Type* categorizes the clinical entry. The *Content* attribute is modality-agnostic; although our local dataset mainly contains text, this schema easily accommodates external image-derived textual features, such as structured radiological findings converted via vision-to-text models, without modifying the underlying data structure.

Based on this schema, we constructed two complementary resources. The *IPF Knowledge Base* integrates progress notes, medical orders, and discharge summaries. Additionally, the *Medication Knowledge Base* covers 72 drugs identified from our dataset and details the dosage, administration, and therapeutic effects of each medication.

### 2.2 IPF-RAG framework architecture

#### 2.2.1 Architecture overview

As illustrated in Figure 2, IPF-RAG features a decoupled, model-agnostic architecture with two core modules: the *Temporal Retrieval Module (TRM)* and the *Structured Enhancement Module (SEM)*. The TRM first retrieves the most relevant, time-aligned clinical cases and pharmaceutical details from the knowledge bases. These retrieved contexts are then routed to the SEM, which formats them into structured prompts that act as dynamic demonstrations for a few-shot learning paradigm. This setup restricts the LLM to generating diagnostic and therapeutic plans that are logically sound and chronologically appropriate. The decoupled input layer enables plug-and-play feature extractors to process medical images such as chest X-rays, thereby demonstrating the framework’s extensibility to incorporate cross-modal clinical data via a vision-to-text pipeline.

**Figure 2:**
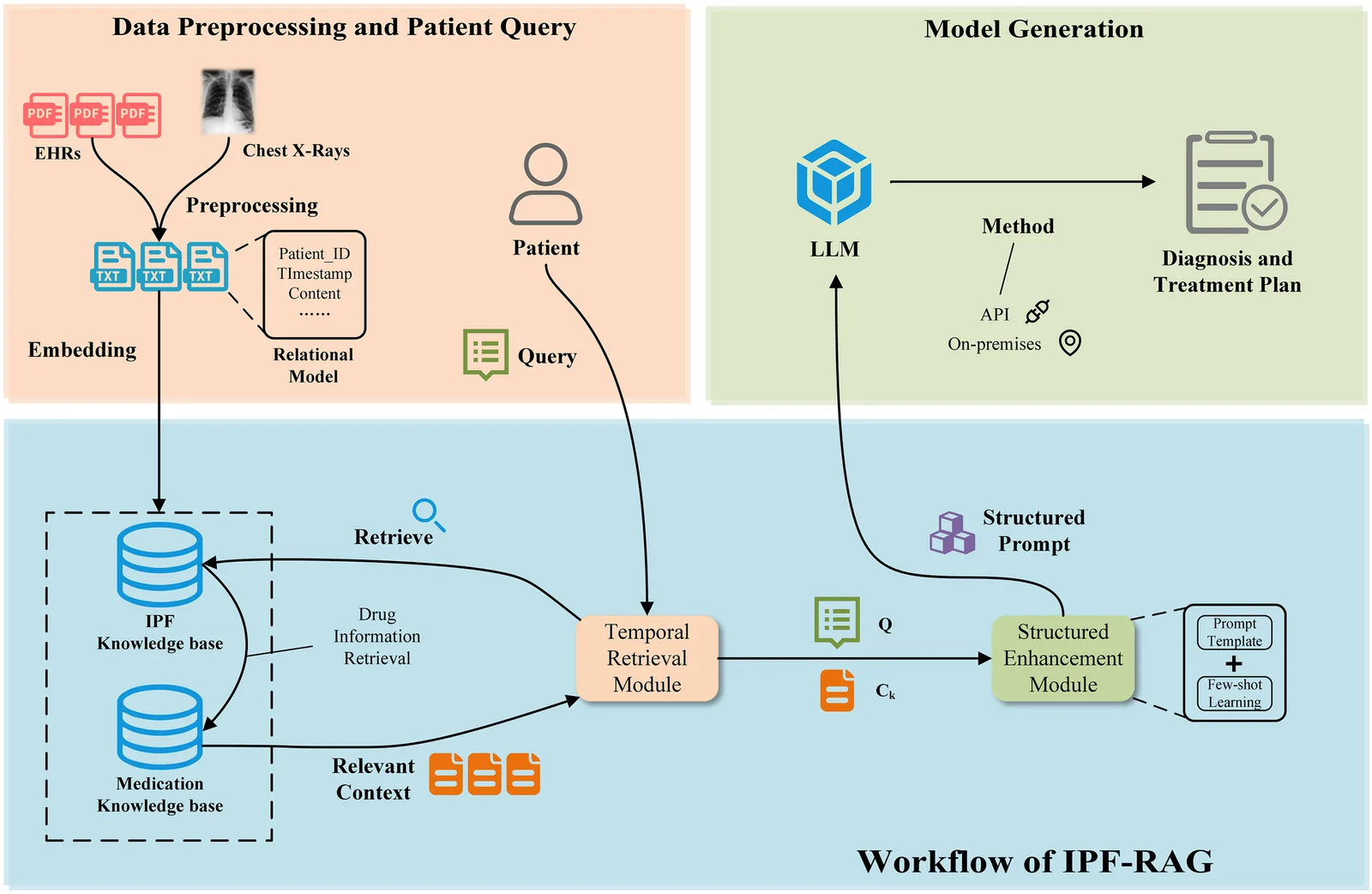
Overview of the IPF-RAG framework. The pipeline spans data preprocessing, query processing with temporal and structural optimization, and response generation.

To leverage the structured schema of our IPF clinical dataset, we propose a three-tiered hybrid retrieval architecture for the TRM, shown in Figure 3. Unlike conventional BM25-based methods (33), this hierarchical design explicitly captures implicit clinical correlations (e.g., between “bilateral lung crackles” and “pulmonary fibrosis”) and preserves temporal dependencies through three sequentially coupled layers.

**Figure 3:**
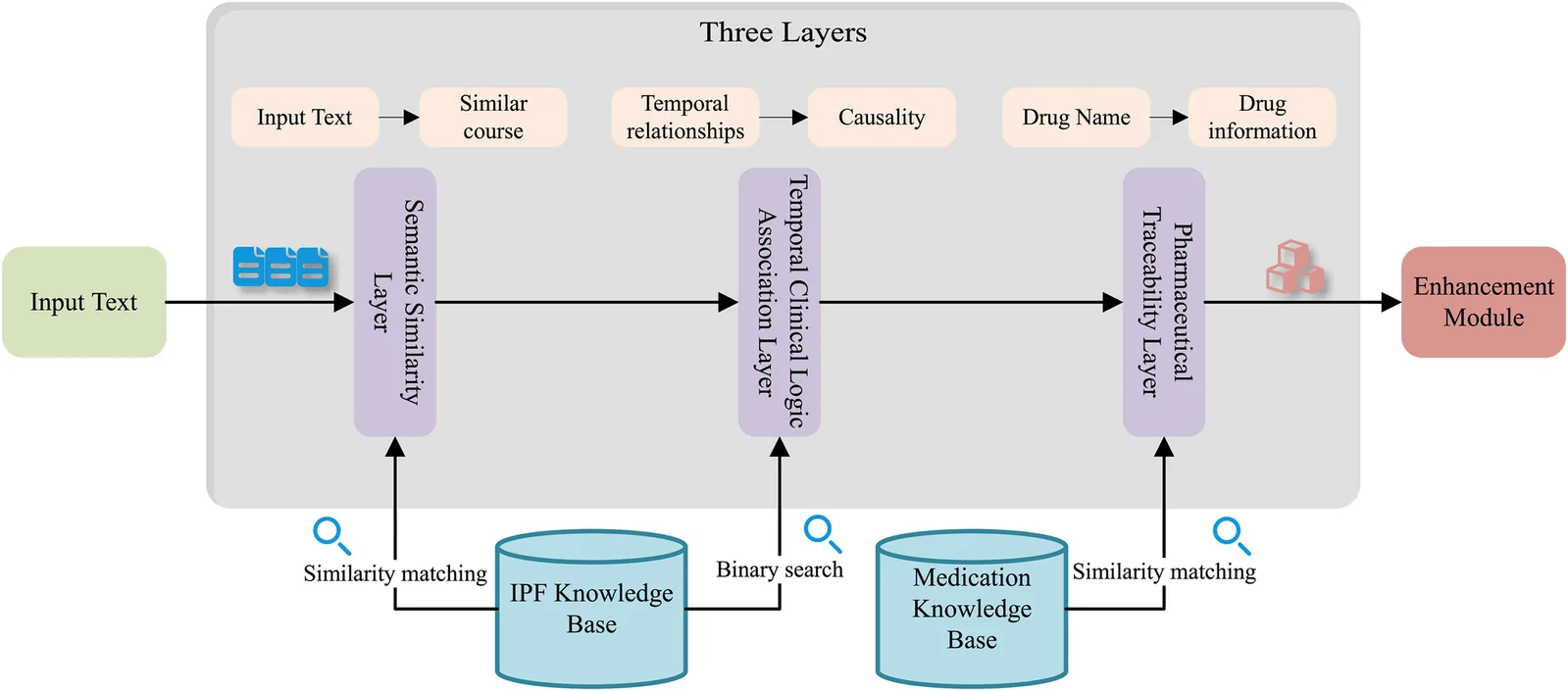
Flow chart of a three-level retrieval algorithm for IPF. The architecture sequentially couples a semantic similarity layer, a temporal clinical logic association layer, and a pharmaceutical traceability layer to preserve temporal dependencies and map clinical notes to specific medical orders.

#### 2.2.2 Temporal retrieval module

**Semantic Similarity Layer:** This layer interfaces with the vectorized IPF knowledge base via a domain-specific text embedding model, to identify historical clinical notes similar to the target patient’s current symptoms. For this step, we used FAISS (34) for efficient large-scale nearest-neighbor retrieval.

**Temporal Clinical Logic Association Layer:** Once similar progress notes are identified, this layer aligns the matched notes with corresponding medical orders. Since clinical notes and medical orders are often recorded asynchronously in real-world EHRs, we adopt temporal proximity matching rather than exact timestamp equivalence for this alignment. Because chronological timestamps increase monotonically within each patient’s records, we treated temporal alignment as a nearest-neighbor search problem over a sorted array. We implemented a binary search algorithm using the retrieved note’s timestamp as the search key, reducing the temporal localization complexity from the O(N) of exhaustive sequential scanning to O(logN). We also applied a dynamic sliding time-window strategy to capture adjacent medical orders, ensuring the completeness of the retrieved regimens.

**Pharmaceutical Traceability Layer:** Finally, this layer cross-references the retrieved medical orders against the medication knowledge base to extract granular pharmacological details. Adding these precise clinical constraints to the generation process improves the clinical safety and credibility of the proposed treatment plans.

#### 2.2.3 Structured enhancement module

The temporally informative clinical cases retrieved by the TRM are fed directly into the SEM. Because specialized clinical datasets for rare diseases such as IPF are typically limited in annotated scale, we design this module based on a few-shot in-context learning paradigm (35, 36) to achieve task adaptation via a minimal set of high-quality examples. The SEM imposes explicit logical constraints on LLM generation by formatting the retrieved cases into dynamic in-context demonstrations. Each demonstration consists of an exemplar input-output pair drawn from real-world clinical records. Embedding these demonstrations alongside the target patient’s current symptoms and clinical status into a structured medical prompt template shown in Figure 4 conditions the model to focus on key clinical decision points. This alignment of the reasoning chain with real clinical practice reduces the hallucination risks common in unconstrained LLM generation.

**Figure 4:**
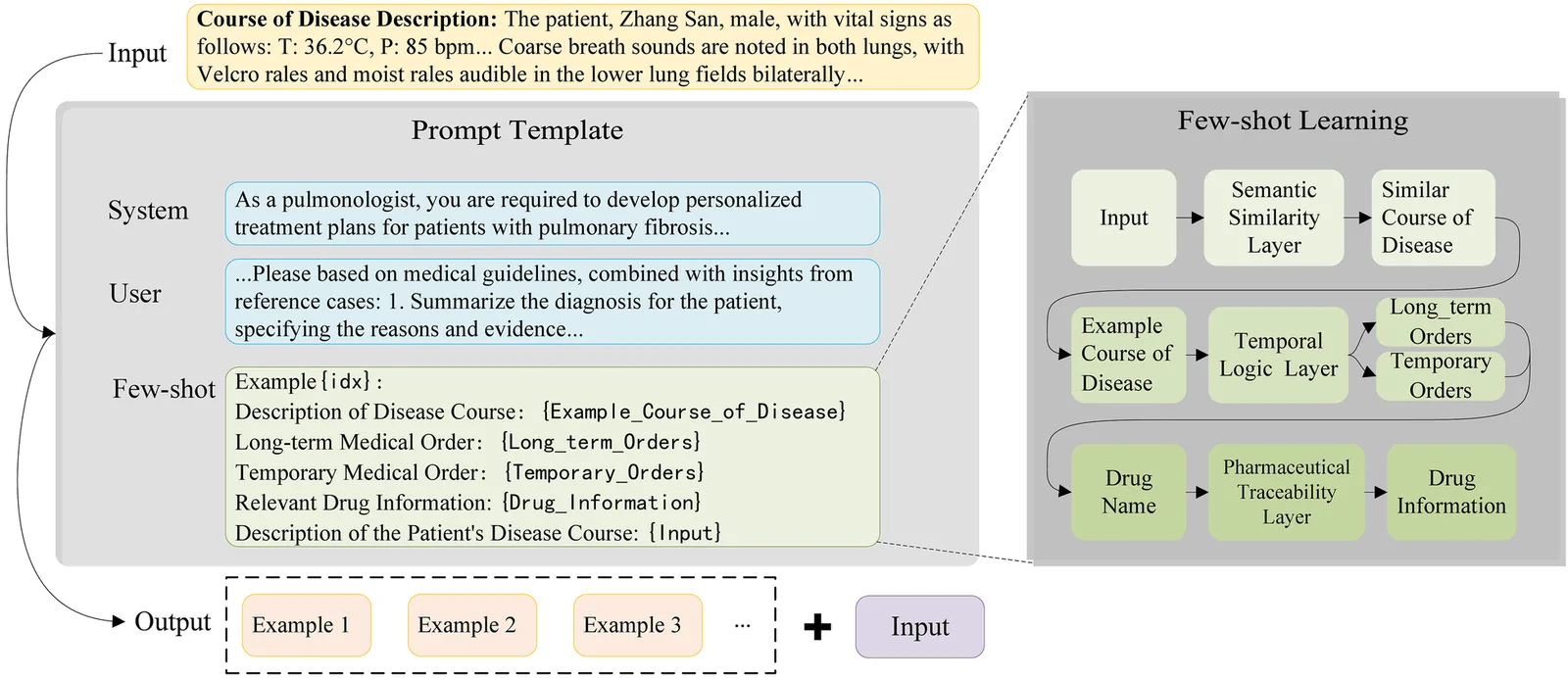
Few-shot prompt template designed for the Structured Enhancement Module (SEM). The template integrates the temporally retrieved clinical cases alongside the target patient’s current symptoms, enforcing medical logic constraints to guide the LLM’s diagnostic reasoning.

## 3 Results

### 3.1 Experimental setup

#### 3.1.1 Evaluation dataset

To evaluate the diagnostic performance of the RAG framework, we used a dataset containing 58 IPF patient trajectories. These patients were partitioned at a 70:30 ratio into a retrieval knowledge base and an evaluation set. Although the specialized medical domain limits the sample size, the test set provides extensive clinical context. By manually extracting admission details such as baseline status and medical history, we constructed a comprehensive set of evaluation prompts. This benchmark was verified by experts to simulate clinical intake, ensuring an objective framework assessment.

To mitigate single-center data limitations and evaluate framework generalizability, we conducted a cross-cohort external validation using the publicly available MIMIC-IV (37) database. We aligned clinical texts from the MIMIC-IV-Note (38) discharge table with corresponding chest radiographs from MIMIC-CXR-JPG (39) using unique patient and admission identifiers. We constructed a test set of 50 randomly selected cases from the remaining eligible cohort after initially identifying 187 IPF-related patients with standard-view radiographs and excluding those without available discharge notes. To accurately assess the framework’s capability to integrate vision-derived features and simulate resource-constrained clinical settings where expert radiological reports are delayed or unavailable, all existing radiological findings were masked in the textual records.

#### 3.1.2 Implementation details

Prior to experimentation, all data and knowledge bases were fully anonymized. All core LLM inference was performed via API calls. For the primary evaluations of the proposed IPF-RAG framework and baseline comparisons, we evaluated three standard large language models. These included the foundation model DeepSeek-V3 (40) and two inference-optimized models, specifically DeepSeek-R1 and GPT-o3 (41). For text embeddings, we applied the bge-small-zh-v1.5 model from BAAI. We selected this model for its optimization in Chinese semantic processing to support both vector database construction and retrieval tasks. To maintain high-precision context extraction, the retrieval hyperparameter was uniformly set to top-k=1 across all RAG-enabled configurations in the primary experiments. We additionally evaluated k=1–5 in a separate sensitivity analysis to examine the effect of retrieving multiple historical cases on generation performance.

For the external validation tasks, we relied on DeepSeek-V3 as the base language model. Instead of training a proprietary vision-language model, we used Gemini 3.1 Pro (42) as a decoupled vision-to-text bridging model to extract structured pathological features directly from the raw CXR images. Prior work has validated this approach for CXR feature extraction (43). We then fed these visual features and the masked clinical texts into DeepSeek-V3 to query our entire IPF Knowledge Base. Finally, GPT-o3 functioned as the automated evaluator for the generated outputs.

#### 3.1.3 Baselines

We evaluated our IPF-RAG framework on the primary text-based tasks by comparing it with three baseline categories. For a fair comparison, we tested all baselines using the three language models described previously. The baselines include:

- **General-Purpose LLMs:** These are the base models without any RAG enhancement.
- **Standard RAG:** This baseline performs conventional semantic retrieval over the IPF knowledge base and directly incorporates the top-k retrieved chunks into the prompt. For a fair comparison, Standard RAG is also allowed to access the same pharmaceutical knowledge base as IPF-RAG, with medications mentioned in the retrieved chunks exactly matched to corresponding drug information and added to the prompt. Standard RAG uses the same generation model and settings as IPF-RAG, but does not apply the TRM or SEM.
- **Multi-Query RAG (44):** This retrieval-enhanced baseline reformulates the original query into multiple variants, performs semantic retrieval for these queries, and aggregates the retrieved evidence for generation, thereby broadening retrieval coverage beyond conventional single-query retrieval. We chose this baseline because it shows strong performance in medical information retrieval tasks.

We selected these baselines to validate the effectiveness of the IPF-RAG framework and to compare its performance across foundation and inference-optimized models. This comparison also shows how well IPF-RAG improves the diagnostic and treatment plans generated by the DeepSeek and GPT series on the Chinese dataset.

#### 3.1.4 Quality evaluation metrics

We established a hierarchical quality evaluation framework with three primary dimensions ranked by clinical importance. These dimensions contain specific criteria labeled for later analysis. The first is *A. Clinical Safety*, which includes A1: Risk Minimization and A2: Warnings & Monitoring. The second is *B. Medical Accuracy*, consisting of B1: Medical Logic and B2: Evidence-Based Medicine. The third is *C. Clinical Utility*, covering C1: Clarity & Actionability and C2: Personalization Level. We rated all criteria on a 5-point Likert scale using two complementary assessment methods:

- **Automated Evaluation:** We used GPT-o3 as an automated physician evaluator. It scored model outputs against criteria A1 through C2 to draw on the diagnostic capabilities of advanced language models.
- **Human Evaluation:** To ensure reliability, at least two physicians reviewed each case in a two-round process. In the first round, they scored a random subset to compare the base models with IPF-RAG. In the second round, they applied a preference selection mechanism to compare IPF-RAG with the remaining baselines across the entire dataset.

### 3.2 Evaluation results and analysis

#### 3.2.1 Analysis of automated evaluation results

Table 1 presents the automated evaluation results. Among the foundation models, GPT-o3 consistently outperforms DeepSeek-R1 and DeepSeek-V3 across all configurations. Standard RAG improves upon the base models, but Multi-Query RAG does not offer further improvements. Generating query variations introduces semantic noise, lowering the signal-to-noise ratio during retrieval from highly specialized medical databases.

**Table 1:** Automated evaluation results (1–5 points).

Method	Model	A1	A2	B1	B2	C1	C2	Avg	Δ Avg (95% CI)
General-Purpose LLM	DeepSeek-V3	3.56	3.06	3.33	2.50	3.78	2.89	3.19	−
	DeepSeek-R1	3.83	3.67	3.72	3.11	3.94	3.44	3.62	−
	GPT-o3	4.22	4.22	4.33	3.78	4.44	4.11	4.19	−
Standard RAG	DeepSeek-V3	3.78	3.56	3.72	3.56	3.89	3.56	3.68	Ref.
	DeepSeek-R1	3.78	3.67	3.78	3.61	3.94	3.56	3.72	Ref.
	GPT-o3	4.22	4.11	4.17	4.17	4.50	4.17	4.22	Ref.
Multi-Query RAG	DeepSeek-V3	3.50	3.33	3.39	3.11	3.78	3.44	3.43	−
	DeepSeek-R1	3.72	3.61	3.72	3.67	3.94	3.67	3.72	−
	GPT-o3	4.11	4.11	4.17	4.17	4.39	4.17	4.19	−
IPF-RAG	DeepSeek-V3	4.06	4.11${}^{*}$	4.11	4.33 ${}^{**}$	4.17	3.83	4.10${}^{**}$	+0.43 [0.16, 0.69]
	DeepSeek-R1	4.17	4.22 ${}^{**}$	4.11	4.33 ${}^{**}$	4.17	4.11${}^{**}$	4.19${}^{**}$	+0.46 [0.22, 0.70]
	GPT-o3	**4.72** ${}^{**}$	**4.78** ${}^{**}$	**4.72** ${}^{**}$	**4.89** ${}^{**}$	**4.72** ${}^{*}$	**4.56** ${}^{*}$	**4.73** ${}^{**}$	+0.51 [0.35, 0.67]

Bold and underlined values indicate the highest and second-highest scores, respectively. IPF-RAG was compared with the corresponding Standard RAG using the paired Wilcoxon signed-rank test. Holm adjustment was applied across the three base-model Avg tests (primary family) and across A1–C2 within each base model (secondary families); significance markers use Holm-adjusted p values: ${}^{*}p_{\mathrm{Holm}}<0.05$, ${}^{**}p_{\mathrm{Holm}}<0.01$, and ${}^{***}p_{\mathrm{Holm}}<0.001$. The Δ Avg column reports the paired mean improvement and its 95% confidence interval. The matched-pairs rank-biserial correlations for Avg were $r_{\mathrm{rb}}=0.84$ for DeepSeek-V3, 0.90 for DeepSeek-R1, and 1.00 for GPT-o3, calculated as IPF-RAG minus Standard RAG; positive values favor IPF-RAG and larger absolute values indicate stronger rank dominance.

Our IPF-RAG framework shows statistically significant improvements over all baselines. Compared to the Standard RAG reference, IPF-RAG increases the overall average score across all base models. Specifically, DeepSeek-V3 shows an increase of 0.43 with a 95% confidence interval of 0.16–0.69 and a *p*-value of 0.008. DeepSeek-R1 improves by 0.46, with a confidence interval of 0.22–0.70 and a *p*-value of 0.008. Finally, GPT-o3 achieves an increase of 0.51, with a confidence interval of 0.35–0.67 and a Holm-adjusted *p*-value of 0.0013.

When using GPT-o3 as a representative model to analyze specific clinical metrics, IPF-RAG shows the largest improvements in Evidence-Based Medicine for criterion B2 and Warnings & Monitoring for criterion A2, with Holm-adjusted *p*-values of 0.0019 and 0.0027, respectively. The performance gains in Clarity & Actionability for C1 with a *p*-value of 0.046 and Personalization Level for C2 with a *p*-value of 0.0016 are smaller but remain statistically significant. These findings demonstrate that IPF-RAG improves the readability and individualization of treatment plans, but its main contribution is increasing the clinical safety and evidence-based reliability of the generated responses.

#### 3.2.2 Analysis of human evaluation results

We conducted the human evaluation where each case was reviewed by at least two physicians in a two-stage process. To enable side-by-side comparisons, physicians evaluated treatment plans generated by different RAG types using both Likert scoring and a preference selection mechanism. Table 2 presents the results of the first evaluation round. These results align with the automated findings, showing that the IPF-RAG framework achieves statistically significant improvements over the base models across all configurations. Specifically, IPF-RAG increases the overall average score for DeepSeek-V3 by 0.67, with a 95% confidence interval of 0.47–0.86 and a *p*-value below 0.001. DeepSeek-R1 shows a 0.54 increase with a *p*-value below 0.001. GPT-o3 improves by 0.47, also with a *p*-value below 0.001.

**Table 2:** First round human evaluation results (1–5 points).

Method	Model	A1	A2	B1	B2	C1	C2	Avg	Δ Avg (95% CI)
General-Purpose LLM	DeepSeek-V3	3.17	3.17	3.50	2.92	3.38	3.00	3.19	Ref.
	DeepSeek-R1	3.67	3.88	3.75	3.29	3.79	3.58	3.66	Ref.
	GPT-o3	4.04	3.79	3.96	3.42	3.92	3.75	3.81	Ref.
IPF-RAG	DeepSeek-V3	3.67	3.88	3.79	4.13	3.88	3.79	3.86${}^{**}$	+0.67 [0.47, 0.86]
	DeepSeek-R1	**4.17**	4.21	4.04	4.42	**4.33**	4.04	4.20 ${}^{**}$	+0.54 [0.32, 0.77]
	GPT-o3	4.04	**4.29**	**4.29**	**4.46**	**4.33**	**4.25**	**4.28** ${}^{**}$	+0.47 [0.28, 0.65]

Bold and underlined values indicate the highest and second-highest scores, respectively. General-Purpose LLM serves as the reference for each model. Statistical significance of Avg was evaluated using the paired Wilcoxon signed-rank test with Holm correction across the three model-specific comparisons; ${}^{**}p_{\mathrm{Holm}}<0.01$. The Δ Avg column reports the paired mean improvement with its 95% confidence interval. The Avg rank-biserial correlations were $r_{\mathrm{rb}}=1.00$ for DeepSeek-V3, 1.00 for DeepSeek-R1, and 0.97 for GPT-o3; positive values favor IPF-RAG.

To reduce costs in the second evaluation round, we asked physicians to state their preferences between our method and the remaining baselines. Thirty physicians each evaluated two randomly assigned cases, resulting in 60 case-level physician assessments across the 18-case test set. Figure 5 shows these results. Physicians found that models augmented with our method produced better diagnostic and treatment suggestions than the other baselines. Our method was judged better than Standard RAG in 86.7% of the comparisons, comparable in 8.3%, and worse in 5.0%. Against Multi-Query RAG, it was judged better in 68.3% of the comparisons, comparable in 26.7%, and worse in 5.0%. These manual evaluation results demonstrate that IPF-RAG improves the quality of treatment plans generated by large language models.

**Figure 5:**
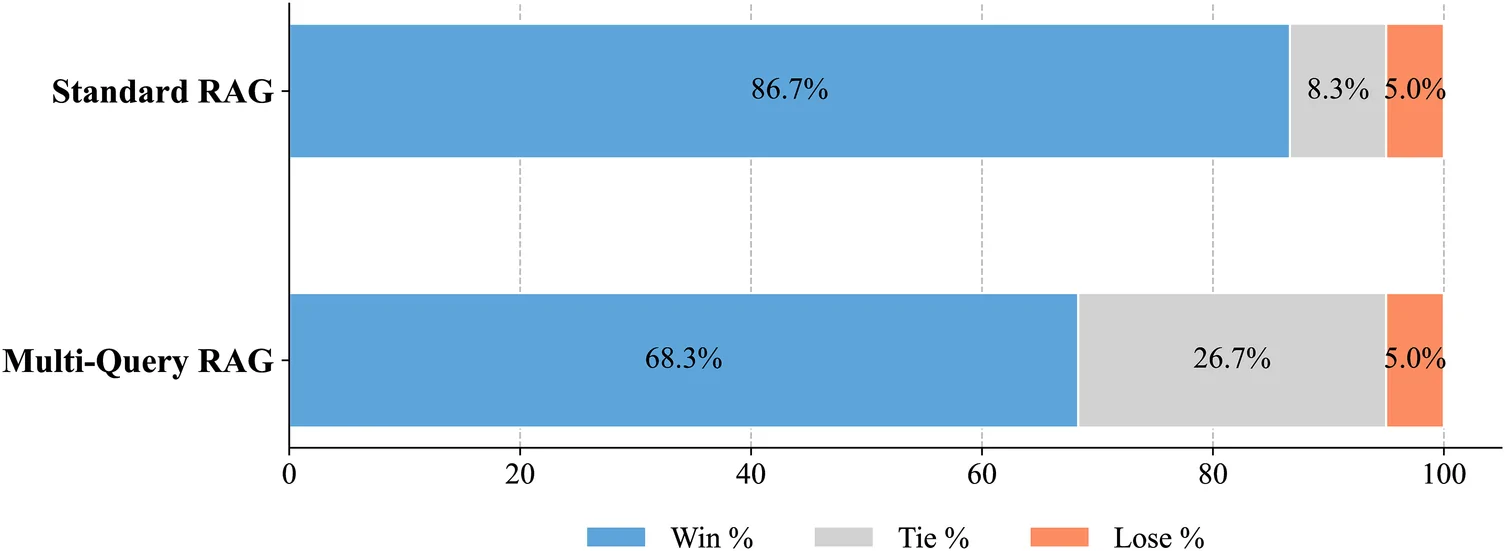
Second round human evaluation results based on physician preference selection. The chart illustrates the win, tie, and loss rates of the proposed IPF-RAG framework against Standard RAG and Multi-Query RAG baselines.

#### 3.2.3 Reliability and consistency analysis of the evaluation framework

To assess the reliability of the evaluation framework, we first examined the internal consistency of the six evaluation dimensions. The automated evaluation scale showed excellent internal consistency (Cronbach’s α=0.934; McDonald’s $\omega_{t}=0.935$). The first-round human evaluation also demonstrated good internal consistency across the six dimensions (Cronbach’s α=0.882; McDonald’s $\omega_{t}=0.885$). Moreover, substantial inter-physician agreement was observed, with a quadratically weighted Gwet’s AC2 of 0.805 (95% CI: 0.769–0.841; p<0.001).

We further assessed the agreement between the automated evaluator, GPT-o3, and expert physicians, as shown in Figure 6. The heatmap in Figure 6(a) shows that the assigned scores had a skewed distribution and were predominantly concentrated in the 3–5 range. This concentration reflects the generally high baseline quality of the generated treatment plans, which rarely received very low ratings.

**Figure 6:**
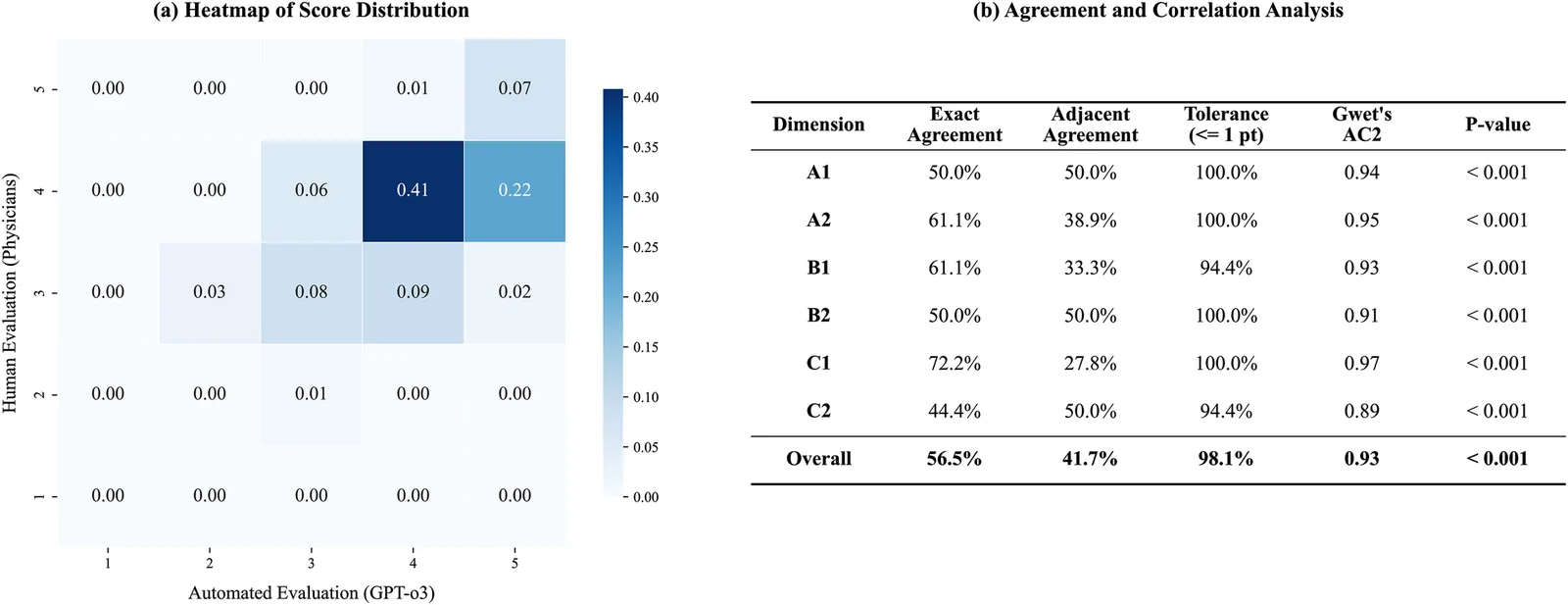
Agreement and reliability analysis between automated (GPT-o3) and human (physicians) evaluations. **(a)** Heatmap of the score distribution across a 5-point Likert scale. **(b)** Detailed inter-rater agreement metrics across clinical evaluation dimensions. To address the skewed score distribution, the table reports Exact Agreement, Adjacent Agreement, Tolerance Agreement (score difference ≤1), and Gwet’s AC2 coefficient with corresponding *P*-values.

Because conventional kappa statistics can be sensitive to prevalence imbalance and skewed ordinal rating distributions, we additionally used tolerance agreement and Gwet’s AC2 to characterize human–AI agreement. As shown in Figure 6(b), the automated evaluator demonstrated substantial agreement with physician assessments across the clinical evaluation dimensions. Taken together, the internal-consistency and inter-rater analyses provide additional evidence supporting the reliability of the proposed evaluation framework.

#### 3.2.4 Ablation study

We conducted an ablation study using DeepSeek-V3 and evaluated the results with GPT-o3 to determine the individual contributions of the Temporal Retrieval Module (TRM) and Structured Enhancement Module (SEM) in our framework. Table 3 shows that the complete IPF-RAG model incorporating both the TRM and SEM achieved the highest performance across all evaluation dimensions with an average score of 4.16. Removing either component caused a statistically significant drop in overall performance, which demonstrates the necessity of both modules.

**Table 3:** Ablation study results.

TRM	SEM	A1	A2	B1	B2	C1	C2	Avg	Δ vs. Full (95% CI)
✓	✓	**3.89**	**4.44**	**4.00**	**3.67**	**4.61**	**4.33**	**4.16**	Ref.
✓		3.22	3.78	3.50	2.89	4.00	3.83	3.54${}^{*}$	−0.62 [−1.18, −0.06]
	✓	3.00${}^{*}$	4.11	3.00${}^{*}$	3.44	4.17	3.28${}^{**}$	3.50${}^{*}$	−0.66 [−1.09, −0.23]
Standard RAG		2.61${}^{**}$	3.67${}^{**}$	2.61${}^{**}$	2.50${}^{**}$	3.83${}^{**}$	3.00${}^{**}$	3.04${}^{**}$	−1.12 [−1.58, −0.66]

TRM: Temporal Retrieval Module; SEM: Structured Enhancement Module. Scores are means on a 5-point Likert scale. Full IPF-RAG serves as the reference for all paired Wilcoxon signed-rank tests. Holm adjustment was applied across the three Avg comparisons (primary family) and across A1–C2 within each reduced configuration (secondary families); significance markers use Holm-adjusted p values: ${}^{*}p_{\mathrm{Holm}}<0.05$, ${}^{**}p_{\mathrm{Holm}}<0.01$, and ${}^{***}p_{\mathrm{Holm}}<0.001$. Δ is calculated as the reduced configuration minus Full IPF-RAG. The matched-pairs rank-biserial correlations for Avg were $r_{\mathrm{rb}}=-0.62$ for w/o SEM, −0.78 for w/o TRM, and −0.93 for Standard RAG; negative values indicate deterioration and larger absolute values indicate stronger rank dominance.

Omitting the TRM to create an SEM-only variant led to a significant overall score decrease of 0.66 with a *p*-value of 0.012. Without the chronological context provided by the TRM, the model showed statistically significant declines in Risk Minimization for criterion A1 ($p_{\mathrm{Holm}}=0.0349$), Medical Logic for B1 ($p_{\mathrm{Holm}}=0.0129$), and Personalization Level for C2 ($p_{\mathrm{Holm}}=0.0046$). This decline indicates that capturing the longitudinal disease trajectory is crucial for generating safe, logical, and personalized treatment plans.

Removing the SEM to test a TRM-only variant also caused a significant drop in overall performance by 0.62 with a *p*-value of 0.021. This overall degradation indicates that, even with accurate temporal retrieval, the SEM contributes to structuring the reasoning process and producing evidence-supported, clinically actionable recommendations. The Standard RAG baseline lacking both modules performed the worst across all metrics, showing a score drop of 1.12 with a Holm-adjusted *p*-value of 0.0016. These findings illustrate the combined value of our dual-module design. The TRM supplies the temporal context needed for safe medical reasoning, while the SEM ensures the output is logically structured, evidence-based, and readable for clinicians.

#### 3.2.5 Analysis of top-k retrieval on treatment generation

Figure 7 uses GPT-o3 scores to compare IPF-RAG and Standard RAG across Top-k settings for k values ranging from 1 to 5. For k>1, the retrieved historical cases were incorporated as separate structured demonstrations in the prompt, following their retrieval ranking. This comparison evaluates the robustness of both methods against varying amounts of retrieved context.

**Figure 7:**
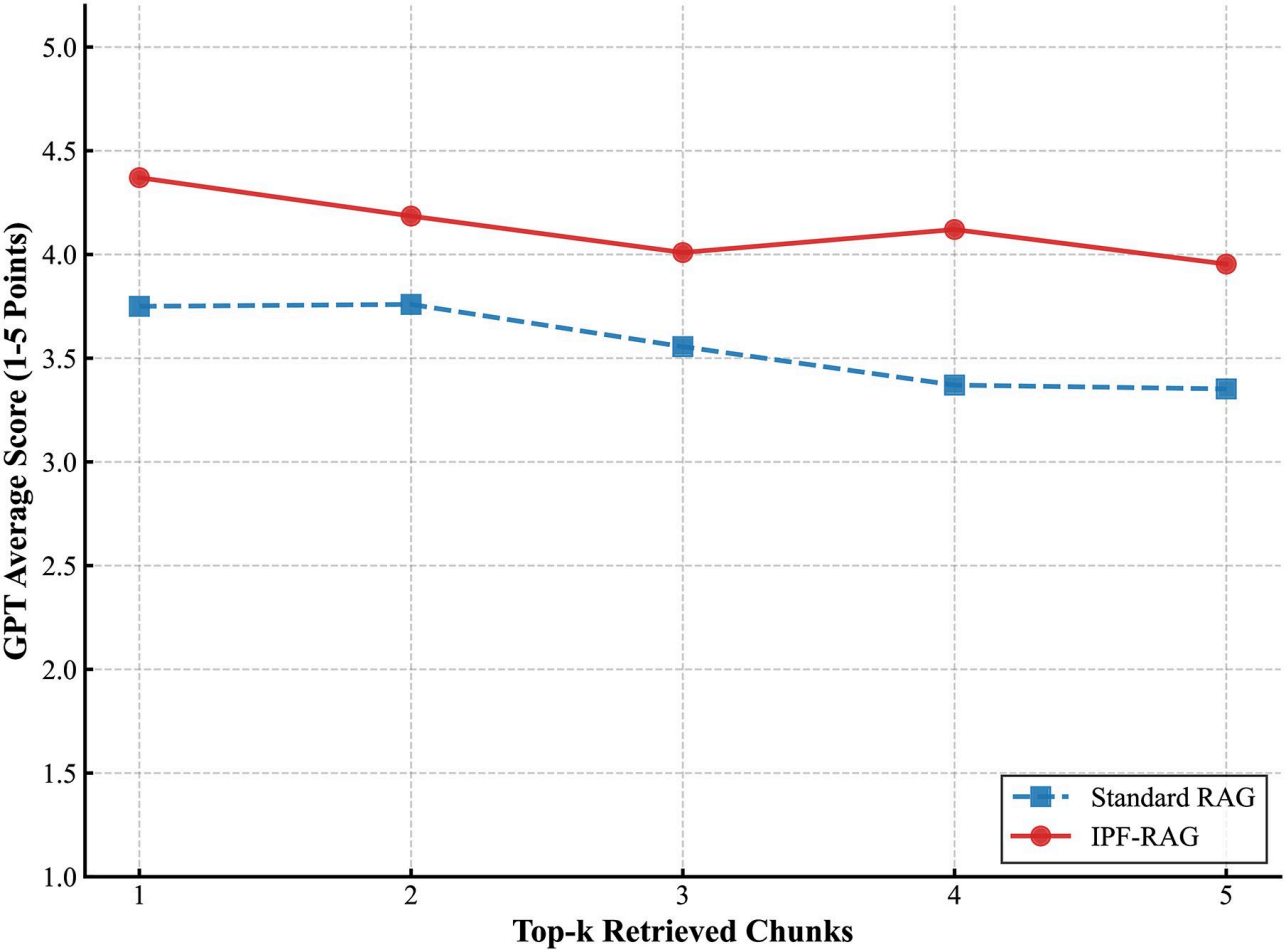
Comparison of Standard RAG and IPF-RAG Performance with Top−k=[1,5]. The vertical axis represents the average evaluation score evaluated by GPT-o3.

The results show a clear performance gap, with IPF-RAG consistently scoring 0.6–0.7 points higher than Standard RAG across all k values. Our method achieves its peak performance at k=1 with a score of approximately 4.37. This early peak shows that temporal-aware retrieval accurately identifies critical evidence without requiring extensive context windows.

As the value of k increases, a distinct divergence in performance trends emerges between the two methods. For Standard RAG, the scores decline monotonically from approximately 3.75 at k=1 to 3.35 at k=5. This degradation likely stems from noise accumulation in unstructured EHRs, because adding retrieved chunks introduces redundant history or irrelevant notes. Without structural filters, the baseline model struggles to separate the signal from the noise and generates hallucinations. In contrast, IPF-RAG maintains high scores above 3.9 across all k values. Organizing the data into temporal sequences prevents information overload and helps the model synthesize multiple pieces of evidence. This stability indicates that our structured framework mitigates interference from irrelevant context and handles clinical data redundancy.

#### 3.2.6 External validation

As detailed in the Experimental Setup, we conducted an external validation on the MIMIC-IV dataset to evaluate the generalizability of the IPF-RAG framework. We integrated raw chest radiographs into masked clinical records using a vision-to-text bridging mechanism to assess the framework’s performance in a zero-shot, cross-cohort setting. We omitted the Multi-Query RAG baseline from this phase because our previous evaluations showed it introduced retrieval noise and performed worse than Standard RAG.

Table 4 shows that the IPF-RAG framework outperformed the baseline methods in this cross-modal and cross-lingual setting. Compared to the Standard RAG baseline, IPF-RAG achieved a statistically significant improvement by increasing the overall average score by 0.50, with a 95% confidence interval of 0.23–0.78 and a Holm-adjusted *p*-value below 0.01. The framework showed strong advantages in Warnings & Monitoring for criterion A2 with a score of 4.46 and Evidence-Based Medicine for B2 with a score of 4.26. The high scores of 4.54 in Clarity & Actionability for C1 and 4.58 in Personalization Level for C2 indicate that our structural and temporal modules successfully synthesize new visual features with retrieved evidence. These findings indicate that IPF-RAG, as a model-agnostic architecture, exhibits promising generalizability in the evaluated tasks, and supports the integration of vision-derived text inputs and external datasets without modifications to its core algorithm framework in the tested scenarios.

**Table 4:** External validation results (1–5 points).

Method	A1	A2	B1	B2	C1	C2	Avg	Δ Avg (95% CI)
General-Purpose LLM	3.82	3.80	3.72	3.22	4.00	3.80	3.73	−
Standard RAG	3.66	3.82	3.58	3.60	4.10	4.08	3.81	Ref.
IPF-RAG	**4.04**	**4.46**	**3.98**	**4.26**	**4.54**	**4.58**	**4.31** ${}^{**}$	+0.50 [0.23, 0.78]

Bold values indicate the higher scores. Standard RAG serves as the reference (Ref.). Statistical significance of Avg was evaluated using the paired Wilcoxon signed-rank test with Holm correction across the two baseline comparisons; ${}^{**}p_{\mathrm{Holm}}<0.01$. The Δ Avg column reports the mean improvement over Standard RAG with its 95% confidence interval. The Avg rank-biserial correlations were $r_{\mathrm{rb}}=0.55$ vs. Standard RAG and 0.61 vs. General-Purpose LLM; positive values favor IPF-RAG.

### 3.3 Case study

Figure 8 presents a case study of an elderly male with “worsening dyspnea for 2 months” and an SpO_2_ decrease to 89%, indicating an acute exacerbation phase.

**Figure 8:**
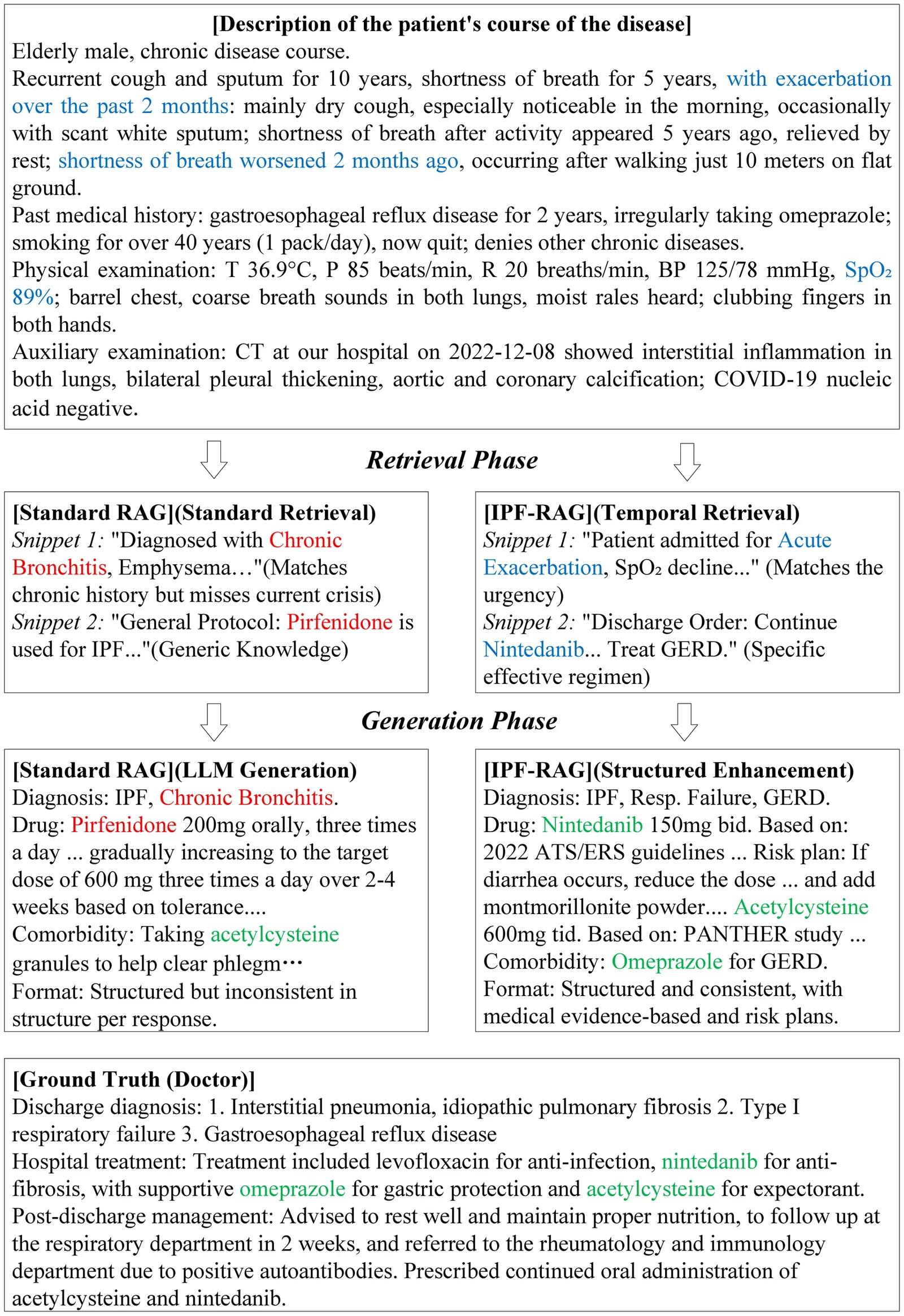
Standard RAG and IPF-RAG responses for the same patient case. **Green** highlight for accurate parts. **Red** highlight for incorrect parts. **Blue** highlight for knowledge parts that help in temporal retrieval of answers.

Standard RAG, relying on static semantic similarity, primarily matched keywords such as “chronic cough” and retrieved unrelated “Chronic Bronchitis” records. By tracking the dynamic progression to hypoxemia, the Temporal Retrieval Module identified a relevant “acute exacerbation” case alongside its clinical medication timeline, prioritizing a specific nintedanib regimen over general recommendations.

Furthermore, standard RAG generated a pirfenidone prescription that deviated from the ground truth. IPF-RAG, however, produced a structured plan aligned with the physician’s decision, recommending nintedanib 150mg bid and including omeprazole for GERD management. These observations suggest that integrating structural constraints may help mitigate factual inaccuracies and support comprehensive comorbidity coverage.

## 4 Discussion

The current study introduces IPF-RAG, an architecture designed to manage the longitudinal complexity of IPF. Evaluation results indicate that the framework effectively enhances LLMs in clinical decision-making, particularly in the dimensions of Evidence-based Medicine and Warning & Monitoring. As demonstrated by the ablation study, these improvements are not merely additive but stem from the synergistic interaction of its core components. The TRM anchors the retrieval process in the patient’s chronological disease trajectory, enabling the model to differentiate acute exacerbations from stable phases—a nuance frequently missed by static keyword matching. Simultaneously, the SEM enforces logical constraints on the reasoning process, ensuring that temporally aligned evidence is translated into safe and actionable medical regimens.

Beyond conventional RAG, Generation-Augmented Retrieval (GAR) provides a complementary strategy for grounding medical language models. By incorporating model-generated information into retrieval and grounding the retrieved evidence in clinical practice guidelines, GAR may improve retrieval relevance and reduce hallucination risk (45). Compared with GAR, conventional RAG offers a more direct evidence-retrieval pipeline but remains sensitive to query quality and retrieval noise, whereas GAR introduces an additional generation-dependent retrieval step to improve query–evidence alignment. These two paradigms are therefore complementary, and integrating GAR-style retrieval refinement with temporal retrieval may be explored in future work.

From the perspective of clinical translation, the framework demonstrates substantial utility as a clinical decision support (CDS) tool. By automatically synthesizing fragmented, longitudinal EHRs into structured drafts, it functions as a “safety net” for flagging contraindications, thereby minimizing the cognitive load on physicians. Crucially, the rigorous consistency analysis using Gwet’s AC2 coefficient revealed an outstanding alignment between the automated evaluator and human experts. The observed agreement between automated and physician evaluations provides additional support for the reliability of the evaluation framework. In addition, the inclusion of Clarity & Actionability as a clinical utility dimension is consistent with recent evaluations of LLM-generated medical texts, which have emphasized quality, readability, and actionability as complementary aspects of practical usefulness (46–48).

A primary concern regarding specialized medical AI frameworks is their restricted generalizability, often stemming from reliance on single-center datasets. We actively addressed this limitation by conducting a cross-cohort, cross-modal external validation using the publicly available MIMIC database. By employing a decoupled vision-to-text bridging mechanism, the framework successfully processed out-of-distribution clinical notes paired with raw chest radiographs, without requiring any modifications to the core retrieval algorithms. While our primary knowledge base is constructed from a specific institutional context, this zero-shot adaptability suggests that the decoupled, model-agnostic architecture possesses a promising degree of generalizability across different clinical environments.

Nevertheless, several limitations warrant objective consideration. First, the framework’s overall efficacy remains intrinsically tied to the precision of the initial text-matching phase; early semantic retrieval failures can lead to cascading errors in downstream temporal alignment. Although k=1 reduced retrieval noise in the primary experiments, relying on a single retrieved historical case may limit evidence diversity for patients with heterogeneous phenotypes or comorbidities. The k=1–5 sensitivity analysis showed that IPF-RAG remained relatively stable when multiple cases were retrieved, but broader multi-case retrieval remains an area for further investigation. Second, the relatively small internal evaluation set may limit the precision and stability of the statistical estimates. Although the paired Wilcoxon signed-rank test does not require a normality assumption and is suitable for paired comparisons in small samples, its results may still be sensitive to individual cases. We therefore complemented the significance tests with Holm-adjusted p-values and rank-biserial correlations to account for multiple comparisons and characterize effect magnitude, respectively. Third, although the external validation on the MIMIC dataset provides preliminary evidence of external adaptability, prospective, large-scale multi-center clinical trials are required to fully ascertain its safety before real-world deployment. Finally, the current protocol for constructing the temporal knowledge base relies on semi-manual curation. Exploring automated and scalable approaches, such as dynamic clinical knowledge graphs, will be a key direction for future research to further optimize retrieval precision and system scalability.

## 5 Conclusion

In this paper, we present IPF-RAG to support longitudinal clinical decision-making in idiopathic pulmonary fibrosis. By integrating the *Temporal Retrieval Module* and the *Structured Enhancement Module*, the framework incorporates chronological disease trajectories and structured medical logic into large language models. Evaluations on our constructed IPF dataset and the external MIMIC-IV dataset show improvements in clinical safety and evidence-based reasoning, demonstrating high agreement with expert physician assessments. Although prospective clinical trials are necessary prior to clinical application, this model-agnostic architecture provides a potential approach for longitudinal decision support in respiratory medicine.

## Data Availability

The datasets analyzed in this study include three publicly accessible controlled-access datasets and one institutional private dataset. The controlled-access datasets, MIMIC-IV (version 3.1; https://doi.org/10.13026/kpb9-mt58 MIMIC-CXR-JPG (version 2.1.0; https://doi.org/10.13026/kpb9-mt58 and MIMIC-IV-Note (version 2.2; https://doi.org/10.13026/1n74-ne17 are hosted on the PhysioNet platform. Access to these datasets is available to credentialed users upon completion of the required ethics training and execution of the applicable data use agreement. The private IPF dataset generated and analyzed during the current study is not publicly available due to patient confidentiality, ethical restrictions, and institutional data governance requirements. Requests to access the de-identified data should be directed to the corresponding author and will be considered subject to approval by the Research Ethics Review Committee of Peking University Shenzhen Hospital and applicable institutional regulations. The custom code used to support the findings of this study is publicly available in the GitHub repository at https://github.com/unimagitian/IPF-RAG.
