# Supplementary material for: IPF-RAG: temporal-aware retrieval-augmented generation for idiopathic pulmonary fibrosis care
Source: Front Digit Health. 2026 Oct 14;8:1883739. doi: 10.3389/fdgth.2026.1883739 (PMC13713474; doi:10.3389/fdgth.2026.1883739)
Supplement: Supplementary file 1 [file Datasheet1.pdf]

Supplementary Material

1 SUPPLEMENTARY EVALUATION AND EXPERIMENTAL DETAILS

1.1 Detailed Physician Evaluation Protocol

Human evaluation was conducted in two rounds to balance fine-grained, multidimensional clinical assessment with the substantial workload associated with physician review. The internal test set comprised 18 patient cases. The first round used a Likert-based scoring protocol and involved 24 physicians, whereas the second round adopted a preference-based comparison protocol and involved 30 physicians. In both rounds, physicians were blinded to the identity of the generation method.

Most participating physicians were resident physicians, with a smaller number of associate chief physicians. Most resident physicians had backgrounds in internal medicine, whereas all associate chief physicians involved in the evaluation specialized in respiratory medicine. When substantial disagreement occurred and a consensus could not be reached directly, the case was reviewed and adjudicated by a chief physician in respiratory medicine with greater clinical experience. The detailed procedures for the two evaluation rounds are described below.

1.1.1 First-Round Likert-Based Evaluation

The first-round evaluation was designed to provide a fine-grained, criterion-level assessment of the generated diagnostic and treatment recommendations and to support the agreement analysis between human and automated evaluations. In this round, outputs generated by IPF-RAG were compared with those generated by the corresponding general-purpose LLMs without RAG augmentation. Standard RAG and Multi-Query RAG were not included in the first round and were instead evaluated separately using the preference-based protocol in the second round.

Each generated response was assessed using the same six clinical dimensions adopted in the automated evaluation: A1, Risk Minimization; A2, Warnings & Monitoring; B1, Medical Logic; B2, Evidence-Based Medicine; C1, Clarity & Actionability; and C2, Personalization Level. Each dimension was independently scored on a 5-point Likert scale, with higher scores indicating better performance.

Because applying the full six-dimensional scoring framework to all model-generated responses across the complete 18-case test set would impose a substantial evaluation burden on physicians, a subset of cases was randomly sampled from the test set for detailed first-round assessment. A total of 24 physicians participated in this round, and each physician evaluated all responses included in the comparison for the assigned case using the six-dimensional framework. The first-round evaluation yielded 24 physician–case assessments covering 12 unique test cases. Some cases were independently evaluated by two or more physicians, allowing inter-physician reliability to be further examined.

For each assigned case, physicians used the same six-dimensional evaluation framework to score all responses included in the first-round comparison.

The physician ratings obtained in this round served two main purposes. First, they provided an independent human assessment of the performance differences between IPF-RAG and the corresponding non-RAG base models. Second, because the same generated responses were also scored by the automated evaluator, these physician ratings enabled direct assessment of agreement between human and automated evaluations.

Internal consistency across the six evaluation dimensions was assessed using Cronbach's α and McDonald's total omega (ω_t). For the ordinal 1–5 ratings, inter-physician agreement was quantified using quadratically weighted Gwet's AC2 with 95% confidence intervals. Agreement between physician and automated ratings was further assessed using agreement-based measures and Gwet's AC2.

When substantial disagreement occurred among physicians in the clinical assessment of the same case, the case was reviewed and adjudicated by a chief physician specializing in respiratory medicine to establish the final clinical judgment.

1.1.2 Second-Round Preference-Based Evaluation

The second-round evaluation was designed to reduce the physician workload associated with repeated six-dimensional Likert scoring while achieving broader coverage of the complete test set. This round specifically compared IPF-RAG with the two RAG-based baseline methods, Standard RAG and Multi-Query RAG.

A total of 30 physicians participated in the second round. Each physician evaluated two randomly assigned cases, resulting in 60 case-level physician assessments. The case-assignment procedure ensured that every case in the 18-case test set was independently evaluated by at least two physicians.

Each case-level assessment included two pairwise comparisons:

1.IPF-RAG versus Standard RAG;
2.IPF-RAG versus Multi-Query RAG.

Accordingly, the 60 case-level assessments yielded a total of 120 pairwise preference judgments, including 60 comparisons between IPF-RAG and Standard RAG and 60 comparisons between IPF-RAG and Multi-Query RAG.

For each pairwise comparison, physicians were asked to judge whether the diagnostic and treatment recommendation generated by IPF-RAG was *better than*, *comparable to*, or *worse than* the corresponding baseline response.

Unlike the first-round evaluation, physicians were not required to provide separate numerical scores for the A1–C2 dimensions in the second round. This simplified preference-based evaluation substantially reduced the assessment burden while enabling repeated physician evaluation across all 18 test cases.

When substantial disagreement occurred among physicians regarding the clinical preference for the same case, the case was reviewed and adjudicated by a chief physician with extensive clinical experience in respiratory medicine.

Preference outcomes were summarized separately for the two baseline comparisons. For IPF-RAG versus Standard RAG, the proportions of *better than*, *comparable to*, and *worse than* judgments were calculated using the corresponding 60 pairwise preference judgments as the denominator. The same procedure was independently applied to the 60 pairwise judgments comparing IPF-RAG with Multi-Query RAG. Therefore, the percentages reported for each baseline comparison were calculated using the corresponding 60 pairwise judgments, rather than the combined total of 120 judgments.

1.2 Automated Evaluation Prompt Template

The original evaluation prompt was written in Chinese. An English-translated and condensed version is presented below. Repeated response placeholders are omitted for brevity. The identities of the generation models and RAG configurations were concealed using anonymous response identifiers.

The prompt template for automated evaluation:

You are an experienced pulmonologist. Based on the provided patient clinical information, independently evaluate the following anonymized diagnostic and treatment recommendations.

Evaluation dimensions
Please score each recommendation on the following six criteria:
A. Clinical Safety
  A1. Does the recommendation follow the principle of risk minimization?
  A2. Does the recommendation provide appropriate warnings and monitoring?
B. Medical Accuracy
  B1. Are the diagnosis and treatment consistent with clinical reasoning?
  B2. Is the recommendation evidence-based and consistent with clinical guidelines?
C. Clinical Utility
  C1. Clarity and actionability.
  C2. Does the recommendation consider patient-specific factors and personalization?

Each criterion is scored from 1 to 5.
Scoring anchors:
1 (Unacceptable): Contains serious hallucinations or medical errors, poses potential risks to patient safety, or is largely irrelevant.
3 (Acceptable): Generally correct, but lacks sufficient detail or personalization, or contains minor non-critical deficiencies.
5 (Excellent): Accurate and well supported by evidence, consistent with current clinical guidelines, and adequately tailored to the patient’s specific clinical characteristics.

Patient case:
{question}

Anonymized responses:
[Response_01]
{response_01}
...

Scoring output:
For each anonymized response, return six scores in the following order:
[A1, A2, B1, B2, C1, C2]
and provide a brief rationale for the assigned scores.

1.3 Retrieval Settings and Parameters

Table S1. Retrieval Parameters.

Component	Parameter	Value
Embedding	model	bge-small-zh-v1.5
Semantic Retrieval	Similarity metric	Squared L2
Semantic Retrieval	FAISS index type	`IndexFlatL2`
TRM	Sliding-window width	3 chunks
TRM	Temporal-gap threshold	2 days
TRM	Matching direction	Forward
TRM	No-match handling	None
TRM	Tie handling	Later-first
TRM	top-k	1
Generation	Temperature	0
Multi-Query	Query Variants	3

Note: Forward indicates that the earliest order after the target timestamp is preferred. None indicates that no order is returned when temporal matching fails. Later-first indicates that the later adjacent record is considered first during window expansion.

Table S1 summarizes the retrieval settings used in our experiments. Unless otherwise specified, the same settings were used across all compared methods.

1.4 Comparison of Retrieved Context Lengths Across RAG Methods

Although all RAG-based methods used top-$k = 1$, their retrieved contexts differed in length because each framework constructs and organizes retrieved evidence differently. We therefore quantified the actual retrieved-context lengths under the settings used in the main experiments, as shown in Table S2.

Table S2. Retrieved-Context Token Statistics.

Method	Mean $\pm$ SD	Median [IQR]
Standard RAG	958 $\pm$ 496	886.5 [738.0–972.8]
Multi-Query RAG	1864 $\pm$ 426	1902.5 [1644–2171]
IPF-RAG	1478 $\pm$ 564	1369 [1224.5–1648]

Note: Values represent retrieved-context tokens per case across the evaluation dataset. SD denotes standard deviation, and IQR is reported as [Q1–Q3]. Tokens were counted using the DeepSeek-V3 tokenizer.

Standard RAG contained 958±496 retrieved tokens per case on average, compared with 1,478±564 for IPF-RAG and 1,864±426 for Multi-Query RAG. IPF-RAG therefore did not receive the largest context; Multi-Query RAG provided approximately 26% more retrieved context than IPF-RAG but nevertheless showed lower generation performance in the main evaluation, indicating that context volume alone did not monotonically determine performance. We retained the native context construction because the temporally associated clinical orders and medication information are integral components of IPF-RAG. Artificially truncating these structured evidence blocks to match the length of Standard RAG could result in semantic information loss and would effectively evaluate a truncated variant rather than the complete framework.

Nevertheless, unequal context lengths remain a potential confounding factor and are acknowledged as a limitation of the current comparison.

2 SUPPLEMENTARY EXPERIMENTAL RESULTS

2.1 Independent Automated Evaluation with Gemini 3.7 Flash

Table S3. Automated Evaluation Results by Gemini-3.7-Flash (1–5 points).

Method	Model	A1	A2	B1	B2	C1	C2	Avg	Δ Avg (95% CI)
General-Purpose LLM	DeepSeek-V3	3.56	3.00	3.39	2.89	3.67	3.00	3.25	–
	DeepSeek-R1	4.11	4.17	4.17	3.72	4.22	4.17	4.09	–
	GPT-o3	4.11	4.11	4.39	4.06	4.39	4.06	4.19	–
Standard RAG	DeepSeek-V3	3.67	3.89	3.61	3.61	4.06	3.44	3.71	Ref.
	DeepSeek-R1	4.06	4.11	4.17	4.11	4.17	4.00	4.10	Ref.
	GPT-o3	4.39	4.44	4.56	4.33	4.78	4.39	4.48	Ref.
Multi-Query RAG	DeepSeek-V3	3.44	3.56	3.50	3.39	4.06	3.56	3.58	–
	DeepSeek-R1	4.06	4.00	4.11	3.83	4.11	4.00	4.02	–
	GPT-o3	4.44	4.61	4.61	4.39	4.72	4.50	4.55	–
IPF-RAG	DeepSeek-V3	3.94	4.00	4.22*	4.17*	4.39	4.06**	4.13**	+0.42 [0.19, 0.64]
	DeepSeek-R1	4.67*	**4.89****	4.78**	4.83**	4.78**	**4.67****	4.77**	+0.67 [0.45, 0.88]
	GPT-o3	**4.89**	4.83	**4.94**	**4.94***	**4.89**	**4.67**	**4.86****	+0.38 [0.22, 0.54]

Note: Bold and underlined values indicate the highest and second-highest scores, respectively. IPF-RAG was compared with the corresponding Standard RAG using the paired Wilcoxon signed-rank test. Holm adjustment was applied across the three base-model Avg tests (primary family) and across A1–C2 within each base model (secondary families); significance markers use Holm-adjusted p values: $^{*}p_{\mathrm{Holm}} < 0.05$, $^{**}p_{\mathrm{Holm}} < 0.01$, and $^{***}p_{\mathrm{Holm}} < 0.001$. The Δ Avg column reports the paired mean improvement and its 95% confidence interval. The matched-pairs rank-biserial correlations for Avg were $r_{\mathrm{rb}} = 0.78$ for DeepSeek-V3, 0.97 for DeepSeek-R1, and 0.92 for GPT-o3, calculated as IPF-RAG minus Standard RAG; positive values favor IPF-RAG and larger absolute values indicate stronger rank dominance.

Across all three base models, IPF-RAG consistently achieved higher average scores than the corresponding Standard RAG configuration (Table S3). The mean improvements were 0.42 points for DeepSeek-V3, 0.67 points for DeepSeek-R1, and 0.38 points for GPT-o3, with all three 95% confidence intervals remaining above zero. The largest improvement was observed with DeepSeek-R1, for which IPF-RAG increased the average score from 4.10 to 4.77. These results indicate that the performance advantage of IPF-RAG is preserved when the generated responses are evaluated by an independent automated evaluator.

2.2 Patient-Level Five-Fold Cross-Validation

Table S4. Patient-Level Five-Fold Cross-Validation Results (1–5 points).

Method	A1	A2	B1	B2	C1	C2	Avg	Δ Avg (95% CI)	r_{rb}
Standard RAG	3.66	3.98	3.34	3.41	3.90	3.43	3.62	Ref.	–
IPF-RAG	**4.05**	**4.28**	**3.98**	**3.86**	**4.29**	**4.10**	**4.09***	+0.47 [0.18, 0.77]	0.42

Note: Results are based on 58 out-of-fold patients. Answers were generated by DeepSeek-V3 and evaluated using a blinded paired assessment by Gemini 3.7 Flash. Bold values indicate the highest scores, and Standard RAG serves as the reference (Ref.). The Δ Avg column reports the paired mean difference between IPF-RAG and Standard RAG with its 95% confidence interval. Avg significance was evaluated using the two-sided Wilcoxon signed-rank test with Holm correction across A1–C2 and Avg; $^{*}p_{\mathrm{Holm}} < 0.05$. The matched-pairs rank-biserial correlation r_{rb} is positive when favoring IPF-RAG.

The Table S4 showed a consistent advantage for IPF-RAG across all six evaluation dimensions. The overall average score increased from 3.62 for Standard RAG to 4.09 for IPF-RAG, corresponding to a paired mean improvement of 0.47 points (95% CI: 0.18–0.77). The positive rank-biserial correlation ($r_{\mathrm{rb}} = 0.42$) further indicates that the improvement was not restricted to a small number of out-of-fold

cases. Together, these results support the stability of the observed performance gain under patient-level resampling.

2.3 Top-k Re-evaluation with GPT-6-Astra Evaluator

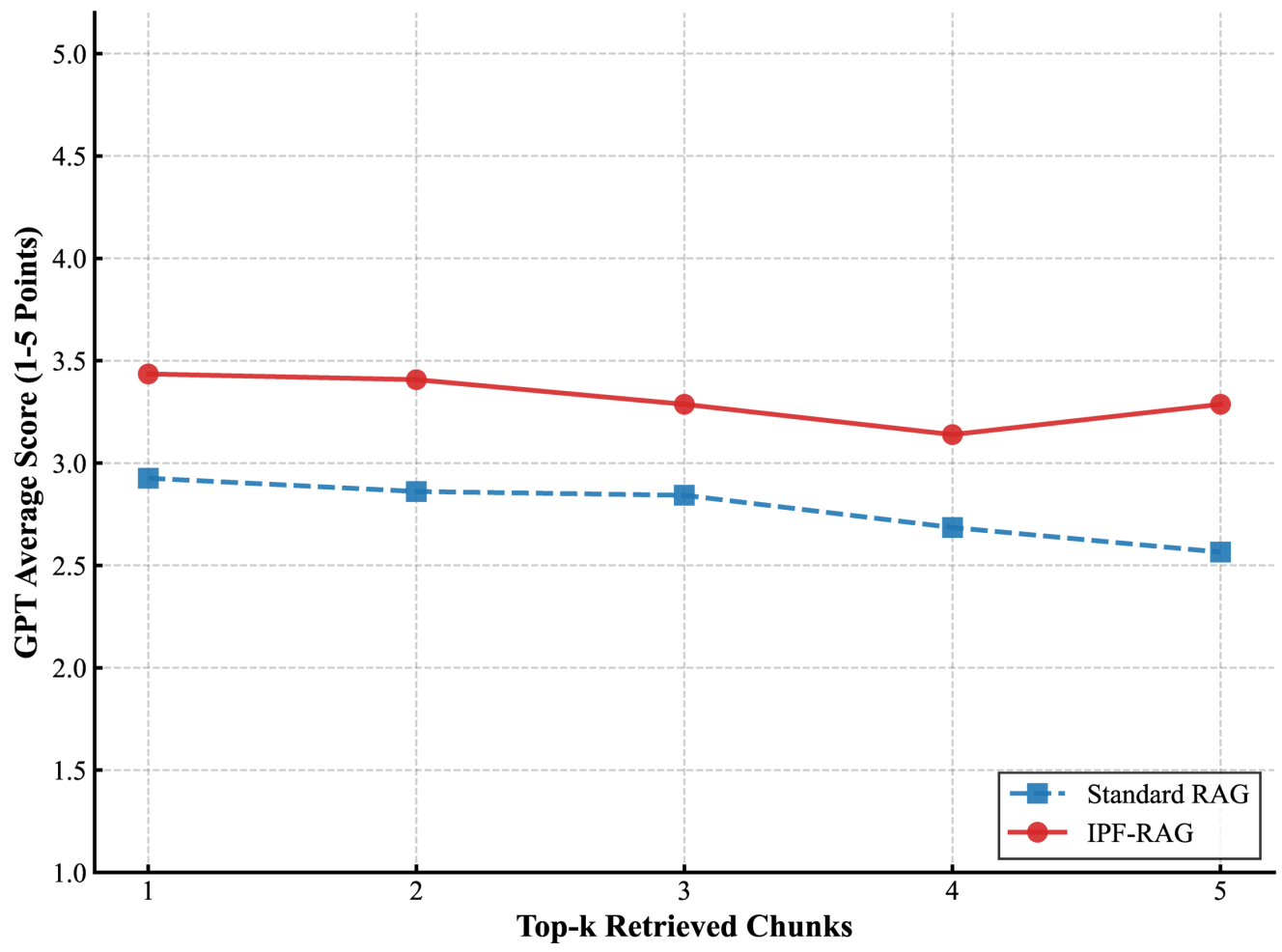

Figure S1. Top-k performance re-evaluated using GPT-6-Astra. The identical DeepSeek-V3-generated responses from the original Top-k experiment were re-evaluated using GPT-6 while all retrieval settings and generated outputs were kept fixed. Although absolute scores were lower than those assigned by GPT-o3, IPF-RAG remained superior to Standard RAG across $k = 1$–5 and the overall Top-k trend remained consistent.

To assess whether the Top-k findings were sensitive to the choice of automated evaluator, we re-evaluated the same set of responses used in the original Top-k analysis with GPT-6. The generated responses were kept fixed, so that the evaluator was the only changed component.

As shown in Fig S1, GPT-6 assigned lower absolute scores than GPT-o3 overall. Nevertheless, the relative pattern remained consistent. IPF-RAG outperformed Standard RAG at every k value, $k = 1$ remained the best-performing setting, and both methods showed an overall tendency toward lower performance as additional retrieved chunks were introduced, with minor local fluctuations.

2.4 Robustness to Different CXR Information Sources

Table S5. Performance under Different CXR Information Sources (1–5 points).

CXR Information Source	Method	A1	A2	B1	B2	C1	C2	Avg	Δ Avg (95% CI)
Model-Generated	Standard RAG	3.66	3.82	3.58	3.60	4.10	4.08	3.81	Ref.
	IPF-RAG	**4.04**	**4.46**	**3.98**	**4.26**	**4.54**	**4.58**	**4.31****	+0.50 [0.23, 0.78]
Original Report	Standard RAG	3.72	4.00	3.54	3.48	4.06	3.84	3.77	Ref.
	IPF-RAG	**4.10**	**4.38**	**4.10**	**4.18**	**4.56**	**4.40**	**4.29****	+0.51 [0.23, 0.80]

Note: Bold values indicate the highest score within each CXR information-source condition; Standard RAG is the reference (Ref.). Avg significance was assessed using two-sided paired Wilcoxon signed-rank tests with Holm correction across two prespecified primary comparisons; $^{**}p_{\mathrm{Holm}} < 0.01$. The Δ Avg column reports the mean paired difference with its 95% confidence interval. Avg rank-biserial correlations were $r_{\mathrm{rb}} = 0.551$ for the model-generated description and $r_{\mathrm{rb}} = 0.536$ for the original radiologist report; positive values favor IPF-RAG.

IPF-RAG showed a similar improvement over Standard RAG under both CXR information-source conditions (Table S5). With model-generated CXR descriptions, the average score increased from 3.81 to 4.31 ($\Delta = +0.50$, 95% CI: 0.23–0.78), whereas using the original radiology reports resulted in a comparable increase from 3.77 to 4.29 ($\Delta = +0.51$, 95% CI: 0.23–0.80). The nearly identical mean improvements and similar rank-biserial effects across the two conditions suggest that the advantage of IPF-RAG is not dependent on the use of model-generated CXR descriptions and remains evident when the original radiology reports are used directly.

3 FAILURE CASE ANALYSIS

Although IPF-RAG improved the overall quality of generated treatment recommendations, it still showed limitations in clinically complex cases involving multiple competing risks. One representative failure case is shown below.

Failure Case: Incomplete Antiplatelet Management After Recent PCI

Clinical Case

An elderly male patient presented with a chronic disease course and an acute exacerbation. He had experienced intermittent chest tightness and dyspnea for more than three months, with recurrence five days earlier and worsening one day before admission. The symptoms occurred after physical activity and were relieved by rest. Approximately two weeks earlier, he had been diagnosed with acute non-ST-segment elevation myocardial infarction (NSTEMI) and had undergone percutaneous coronary intervention (PCI), with two stents placed in the left anterior descending artery (LAD). Five days before admission, he developed chest tightness and shortness of breath after walking approximately 100 meters, accompanied by pain and numbness in the right extremities. One day before admission, the symptoms worsened, and he was unable to lie flat at night. His medical history included diabetes mellitus for more than 20 years, intracerebral hemorrhage eight years earlier, and pulmonary fibrosis for five years. High-resolution computed tomography (HRCT) showed bilateral interstitial pulmonary fibrosis with possible pulmonary hypertension. Echocardiography demonstrated right-heart enlargement and an estimated pulmonary artery systolic pressure of approximately 65 mmHg. Coronary angiography revealed three-vessel coronary artery disease.

Excerpt from the IPF-RAG Response

Generic drug name: Aspirin enteric-coated tablets

- **Dosage and administration:** 100 mg orally once daily.
- **Rationale:** Antiplatelet therapy is essential for coronary artery disease and following PCI.
- **Risk management:** Monitor for bleeding risk, particularly given the patient's history of intracerebral hemorrhage. Monitor for melena and headache, and consider proton-pump inhibitor (PPI) therapy when necessary.

Failure Analysis

Although the response correctly recognized the need for antiplatelet therapy following PCI, IPF-RAG recommended aspirin 100 mg once daily as the only antiplatelet agent. It did not adequately address the need to reassess the early post-PCI antiplatelet strategy in the context of the patient's

recent NSTEMI and two LAD stents, thereby insufficiently accounting for the risk of early stent thrombosis.

Clinical Reference

The patient should first undergo urgent evaluation for recurrent ischemia or heart failure following the recent PCI, including electrocardiography, serial cardiac troponin testing, echocardiography, and assessment for stent-related ischemia. The newly developed right-sided limb symptoms should also prompt neurological examination and cranial imaging. The antiplatelet regimen prescribed at discharge after PCI should be reviewed immediately, with cardiology and neurology consultation to balance the competing risks of stent thrombosis and intracranial bleeding. Aspirin monotherapy should not be initiated or continued without such reassessment. Following ACS treated with PCI, the default antiplatelet strategy generally consists of aspirin combined with a P2Y12 inhibitor, although treatment duration and intensity may be individualized and shortened in patients at high bleeding risk.

This case suggests that the main limitation was not the absence of relevant medical information, but the difficulty of integrating several competing clinical priorities into a single treatment decision. The framework identified a reasonable antiplatelet option and recognized the bleeding risk, but it did not sufficiently weigh this risk against the patient's recent ACS, newly implanted coronary stents, and neurological history. In such situations, recommendations based mainly on retrieved historical patterns may be insufficient when several high-risk conditions must be considered simultaneously.

Our future work will focus on improving risk-aware reasoning rather than simply increasing the amount of retrieved information, by incorporating cross-specialty evidence and guideline-based constraints to better support individualized decisions in cases with competing clinical risks.
